# Oesophageal squamous cell carcinoma: histogram-derived ADC parameters are not predictive of tumour response to chemoradiotherapy

**DOI:** 10.1007/s00330-018-5439-6

**Published:** 2018-05-03

**Authors:** Maiko Kozumi, Hideki Ota, Takaya Yamamoto, Rei Umezawa, Haruo Matsushita, Yojiro Ishikawa, Noriyoshi Takahashi, Tomonori Matsuura, Kei Takase, Keiichi Jingu

**Affiliations:** 1 Department of Radiology, Tohoku Medical and Pharmaceutical University Hospital, 1-12-1 Fukumuro, Miyagino-ku, Sendai, Miyagi Japan; 2 Department of Diagnostic Radiology, Tohoku University Graduate School of Medicine, 2-1 Seiryo-machi, Aoba-ku, Sendai, Miyagi Japan; 3 Department of Radiation Oncology, Tohoku University Graduate School of Medicine, 2-1 Seiryo-machi, Aoba-ku, Sendai, Miyagi Japan

**Keywords:** Oesophageal cancer, Chemoradiotherapy, Magnetic resonance imaging, Diffusion-weighted imaging, Response Evaluation Criteria in Solid Tumours

## Abstract

**Objectives:**

To evaluate correlations between tumour response to definitive chemoradiotherapy (CRT) in oesophageal squamous cell carcinoma (SCC) and histogram-derived apparent diffusion coefficient (ADC) parameters on diffusion-weighted MR images.

**Methods:**

Forty patients with clinical T3–4 oesophageal SCC underwent concurrent CRT. MR examination at 3 T was performed 1–3 days prior to CRT. Readout-segmented echo-planar diffusion imaging was used to acquire ADC maps. Pre- and post-treatment CT examinations were performed. Histogram parameters (mean, 10th, 25th, 50th, 75th, 90th percentiles, skewness and kurtosis) of the ADC values were compared with post-treatment disease status based on RECIST and the tumour regression ratio.

**Results:**

None of the ADC parameters showed significant correlation with post-treatment status (range of Spearman’s ρ values − 0.19 to 0.14, range of *p* values 0.22–0.47) or tumour regression ratio (range of Spearman’s ρ values − 0.045 to 0.18, range of *p* values 0.26–0.96). Neither progression-free survival (PFS) (*p =* 0.17) nor overall survival (OS) (*p =* 0.15) was significantly different between the two groups corresponding to the lower (< median) and upper arms (≥ median) of the mean ADC values.

**Conclusions:**

Histogram-derived pretreatment ADC parameters were not predictive imaging biomarkers for tumour response to CRT in patients with oesophageal SCC.

**Key Points:**

• *Apparent diffusion coefficient (ADC) values are derived from diffusion-weighted MR imaging.*

• *High-resolution diffusion-weighted images are generated by readout-segmented echo-planar diffusion imaging.*

• *Readout-segmented echo-planar diffusion-weighted imaging enabled evaluation of ADC parameters.*

• *Pretreatment ADC parameters do not predict chemoradiotherapy response in patients with oesophageal carcinoma.*

**Electronic supplementary material:**

The online version of this article (10.1007/s00330-018-5439-6) contains supplementary material, which is available to authorized users.

## Introduction

Chemoradiotherapy (CRT) is one of the standard treatment methods for patients with locally advanced oesophageal squamous cell carcinoma (SCC). In patients treated with CRT, the survival rate is lower when the local lesion shows no response in comparison with that in responders [1]. Since CRT is potentially harmful to intact organs such as lungs and kidneys, it is desirable to predict the tumour response to CRT. If the lack of response to definitive CRT can be predicted before treatment in patients with locally advanced oesophageal SCC, CRT can be discontinued and salvage surgery can be scheduled. According to a systematic review, [^18^F]fluorodeoxyglucose positron emission tomography shows heterogeneity in both sensitivity and specificity for prediction of tumour response to neoadjuvant therapy in patients with oesophageal carcinoma [2].

Apparent diffusion coefficient (ADC) values derived from diffusion-weighted imaging (DWI) depend on impediment to free diffusion of water molecules in a single voxel due to the restrictive barriers in tissue compartments [1]. ADC has been used to predict the response to CRT in malignant tumours of other organs [3–7]. Previous studies indicated its potential for predicting treatment response before and during treatment [8–10]. However, the role of ADC measurements on the pretreatment MRI only (before CRT) for predicting tumour response and prognosis in oesophageal carcinoma has not been established. Furthermore, most previous studies only assessed median or mean ADC values, although a few indicated its potential for predicting treatment response using 1.5 T scanners [8, 9]. The study by van Rossum et al. [9] included patients with oesophageal adenocarcinoma and oesophageal SCC; this pathologically heterogeneous cohort may have influenced the baseline ADC values and treatment response to CRT. Other studies by Li et al. [8] and Wang et al. [10] included patients with SCC. However, those studies were conducted using mean or median ADC values. ADC histogram analysis is being increasingly applied for examination of the heterogeneity of diffusion in the tumour region based on pixel distribution [11].

Past studies have shown that DWI is useful for detecting tumours and metastatic lymph nodes in patients with oesophageal carcinoma [12, 13]. ADC calculations based on two *b* values have been well documented in studies of tumours including breast, liver and prostate lesions, as well as in a rat model of glioma [14–17].

Recently, readout-segmented echo-planar diffusion imaging (readout segmentation of long variable echo-trains; RESOLVE) has been developed for clinical use [18]. This technique generates high-resolution diffusion-weighted images with significantly fewer susceptibility artefacts than the single-shot technique. RESOLVE allows a reduced time of echo (TE), which could minimise T2 decay and lead to an improvement in the signal-to-noise ratio (SNR). RESOLVE has demonstrated better image quality than conventional single-shot EPI, especially in regions with strong susceptibility variation [19], and it has been applied for imaging of tumours such as breast and rectal lesions at 3 T [20, 21].

The purpose of this study was to evaluate the correlations between tumour response to definitive CRT in oesophageal SCC and histogram-derived ADC parameters before treatment.

## Materials and methods

The institutional review board approved this prospective study, and written informed consent was obtained from all individual participants included in the study.

### Patients

Forty consecutive patients (36 men, 4 women; mean age, 68.4 years; age range, 51–88 years) with oesophageal SCC who were treated by CRT at our institution between September 2012 and October 2015 were included in this prospective study. Patient and tumour-related characteristics are summarized in Table 1.

**Table 1:** Patient and tumour-related characteristics

Characteristics	*n* (%)
Sex	
Male	36 (90.0)
Female	4 (10.0)
Age (mean ± SD)	68.4 ± 8.5
Location (total)	
Cervical	5
Proximal third	11
Middle third	26
Distal third	13
Gastro-oesophageal junction	4
cT	
cT3	27 (67.5)
cT4	13 (32.5)
Dose (Gy)	
54	1 (2.5)
59.6	2 (5.0)
60	34 (85.0)
61.2	1 (2.5)
62.4	1 (2.5)
64	1 (2.5)
Chemotherapy	
Cisplatin/5-FU	29 (72.5)
Nedaplatin/5-FU	9 (22.5)
Docetaxel/cisplatin/5-FU	2 (5.0)

*5-FU* 5-fluorouracil

The eligibility criteria were (a) oesophageal SCC histologically proven by upper endoscopic biopsy, (b) clinical stage T3 or 4 defined on primary staging chest and abdominal CT scans, (c) no prior chemotherapy, radiotherapy or surgical treatment, (d) no distant metastasis and (e) no other active cancer. The clinical stage grouping was in accordance with the TNM Classification of Malignant Tumours, 7th Edition (UICC).

### MR imaging protocol

A 3-T whole body scanner (MAGNETOM Trio, A Tim System, Siemens Healthcare GmbH, Erlangen Germany) was used for MR imaging. All patients were imaged in the supine position. MR examination was performed within 1–3 days prior to initiation of CRT. MR imaging protocols included transverse T2-weighted imaging (2450/102 [repetition time in milliseconds/echo time in milliseconds], field of view, 272 mm × 340 mm; matrix, 179 × 320; section thickness, 5 mm; slice gap, 0.5 mm; number of slices, 24; acquisition time, 29 s) and readout-segmented echo-planar diffusion-weighted imaging (RESOLVE, prototype sequence, 4500 ms/69 ms, field of view, 220 mm × 320 mm; acquisition matrix, 132 × 192; section thickness, 5 mm; slice gap, 0.5 mm; number of segments, 3; number of slices, 24; acquisition time, 4 min 13 s; and application of motion probing gradient pulse along the x, y and z directions, with *b* values of 50 and 800 s/mm^2^, signal averaging = 3).

### CT imaging protocol

A 64-row-detector CT scanner was used for pre- and post-CRT CT imaging (field of view, 500 mm × 500 mm and 600 mm × 600 mm; tube voltage, 120 kVp; section thickness, 1–2.5 mm). Iodine contrast material was administered intravenously with a 90-s delay.

Pretreatment CT was performed at a median time of 3 days (range, 1–18 days) prior to CRT. Post-treatment CT was performed at a median duration of 1 month (range, 0–3 months) after CRT. Several patients underwent CT, at the discretion of the attending physicians, immediately after the completion of CRT.

### MR image interpretation

**Fig. 1:**
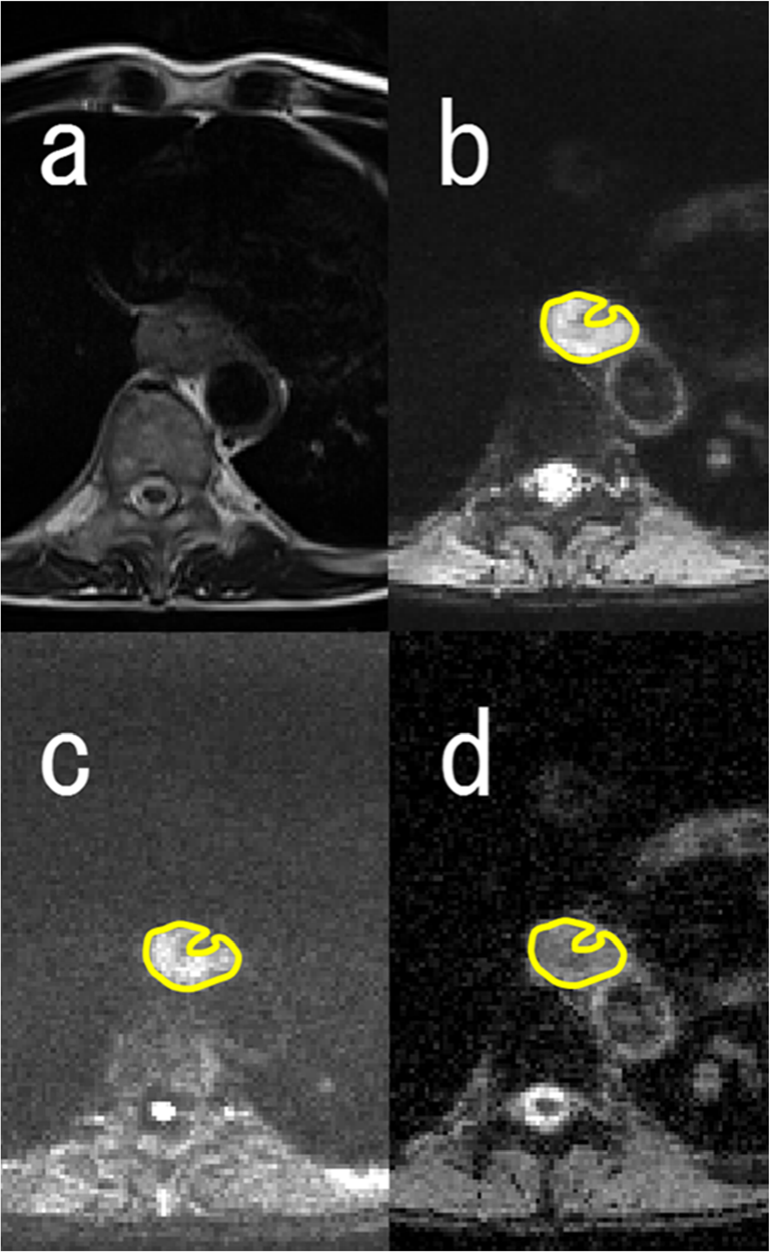
Axial MR images. **a** T2-weighted image, **b** diffusion-weighted image (DWI) with *b* = 50 s/mm^2^, **c** DWI with *b* = 800 s/mm^2^ and **d** apparent diffusion coefficient (ADC) map of a 62-year-old man with oesophageal squamous cell carcinoma. Region of interest (ROI) placement on DWI with *b* = 50 s/mm^2^ (**b**) was performed by referring to the outline of the tumour that showed intermediate to high intensity with oesophageal wall thickening on T2-weighted images (**a**). Each ROI was placed to cover the entire primary lesion without the lumen and was transferred to the corresponding ADC map (**d**)

Two board-certified radiologists with 12 years’ experience of body imaging and 4 years’ experience of radiation oncology evaluated the MR images by consensus reading. They were blinded to subjects’ clinical information and CT images. MR imaging data for ADC maps were transferred to a personal computer and processed with Image J software (http://rsb.info.nih.gov/ij/). All region of interest (ROI) placements on DWI with *b* = 50 s/mm^2^ were decided by the two readers referring to the outline of the tumour that showed intermediate to high intensity with oesophageal wall thickening on T2-weighted images. Each ROI was placed to cover the entire primary lesion without the lumen in multiple slices (in craniocaudal direction also) and was transferred to the corresponding ADC map (Fig. 1).

Histogram analysis of all pixel-based data from ADC maps of the primary lesion was performed. Histogram parameters (mean, 10th, 25th, 50th, 75th, 90th percentiles, skewness and kurtosis) of ADC values were obtained from ROIs placed on the ADC maps for each patient.

### CT image interpretation

The therapeutic effect was assessed according to the Response Evaluation Criteria in Solid Tumours (RECIST: complete response [CR], partial response [PR], progressive disease [PD] and stable disease [SD]) [22]. The major axis of the primary lesion was measured on a slice that showed the largest tumour diameter. CR was defined as disappearance of the primary lesion, PR was defined as at least a 30% decrease in the major axis of the primary lesion, PD was defined as at least a 20% increase in the major axis of the primary lesion and SD was defined as disease other than CR, PD and PR.

### Chemoradiotherapy

All patients were treated with definitive CRT. The daily fractional dose of radiotherapy was 1.8–2.0 Gy, administered 5 days a week, and the total dose was 54–64 Gy. Radiotherapy was delivered with 6–15 MV X-rays up to a dose of 36–40.8 Gy. The radiation fields were designed initially to encompass the primary lesion, metastatic lymph nodes and regional lymph nodes. After that, a 14–24-Gy boost was subsequently delivered, with care taken to avoid the spinal cord, to the primary lesion with a 3-cm margin for the craniocaudal direction and to metastatic lymph nodes with a 1-cm circular margin.

All patients received chemotherapy concurrently during radiotherapy. Two of the six patients with cervical oesophageal carcinoma received an intravenous injection of two cycles of 5-fluorouracil (5-FU) at a dose of 1000 mg/m^2^/day on days 1–5 and 29–33, docetaxel at a dose of 50 mg/m^2^ on days 2 and 30 and cisplatin at a dose of 60 mg/m^2^ on days 2 and 30. Twenty-nine patients received intravenous injections of 1–2 cycles of cisplatin at a dose of 50–70 mg/m^2^ on day 1 (and day 29) and 5-FU at a dose of 700 mg/m^2^/day on days 1–4 (and days 29–32). One patient, for whom neoadjuvant chemoradiotherapy and surgical treatment were planned, received an intravenous injection of cisplatin at a dose of 40 mg/m^2^ on days 1 and 8 and 5-FU at a dose of 400 mg/m^2^/day on days 1–5 and 8–12. At a dose of 30 Gy, the patient’s tumour was judged to be unresectable by a surgeon. Therefore, the patient continued to receive radiotherapy up to a total dose of 60 Gy in 2-Gy fractions. One patient with chronic heart failure, three patients with poor performance status and five patients over 75 years of age received an intravenous injection of 1–2 cycles of nedaplatin at a dose of 50–100 mg/m^2^ on day 1 (and day 29) and 5-FU at a dose of 350-500 mg/m^2^/day on days 1–5 (and days 29–33).

### Statistical analysis

Continuous variables are expressed as mean ± standard deviation, and dichotomous variables are expressed as number and percentage. The patients’ demographic characteristics (sex, age and clinical stage) were analysed using Fisher’s exact test or *t* test. We had small counts (< 5) in the cross-tables for sex and clinical stage. We applied the *t* test to compare the distribution of age because it was normally distributed. The difference in tumour diameter between pretreatment CT and post-treatment CT was evaluated using the paired *t* test. The histogram parameters of ADC values were compared with post-treatment disease status based on RECIST (CR, PR, SD and PD) using the Mann–Whitney *U* test. Spearman rank correlations were used to assess the relationships between ADC parameters and post-treatment status and to compare ADC parameters and the tumour regression ratio. Overall survival period was calculated from the date of starting radiotherapy to death or last follow-up. The probabilities of progression-free survival (PFS) and overall survival (OS) were estimated by the Kaplan–Meier method, and the log-rank test was used for comparison. Statistical analyses were performed using JMP Pro 13 software (SAS Institute, Cary, NC). A *p* value of less than 0.05 was defined as statistically significant.

## Results

**Fig. 2:**
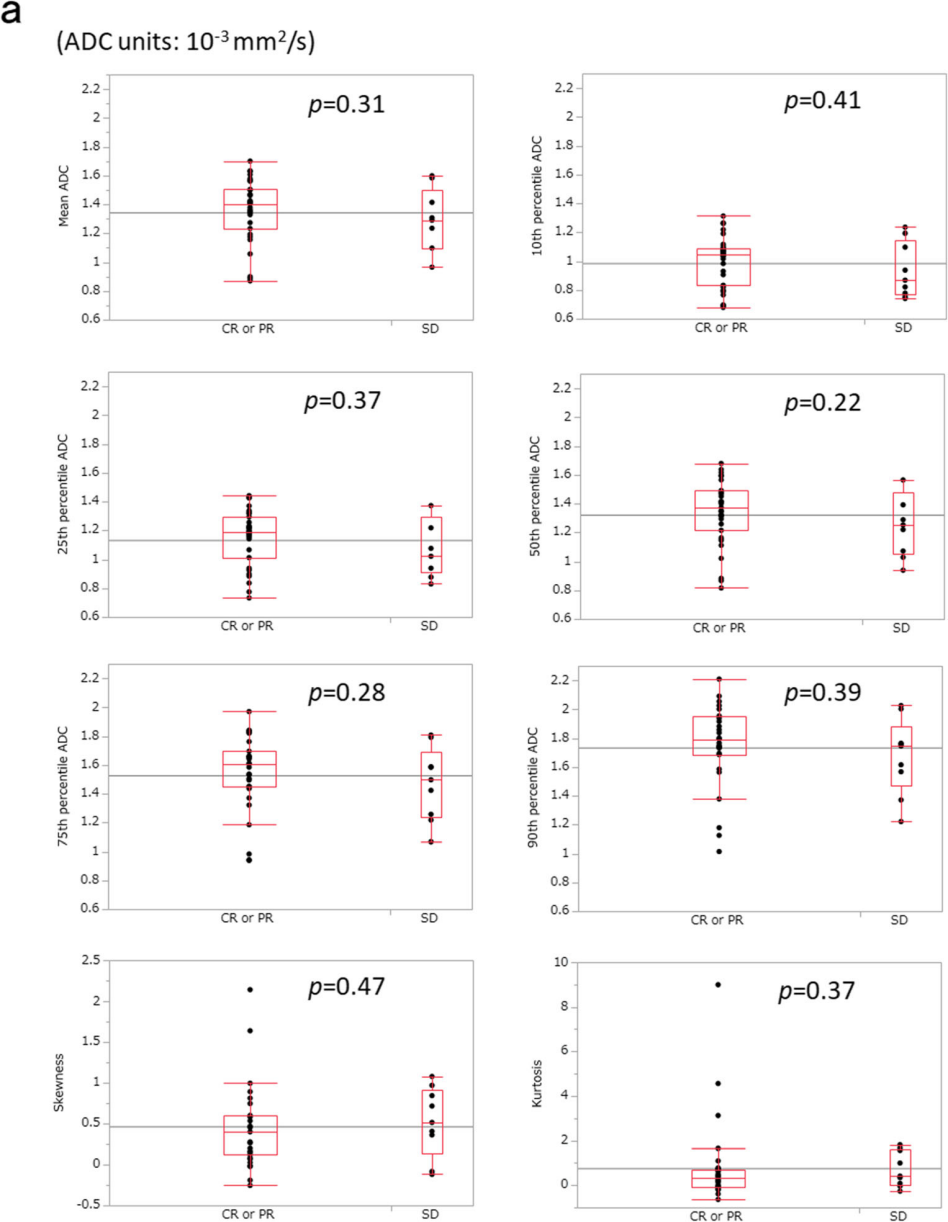

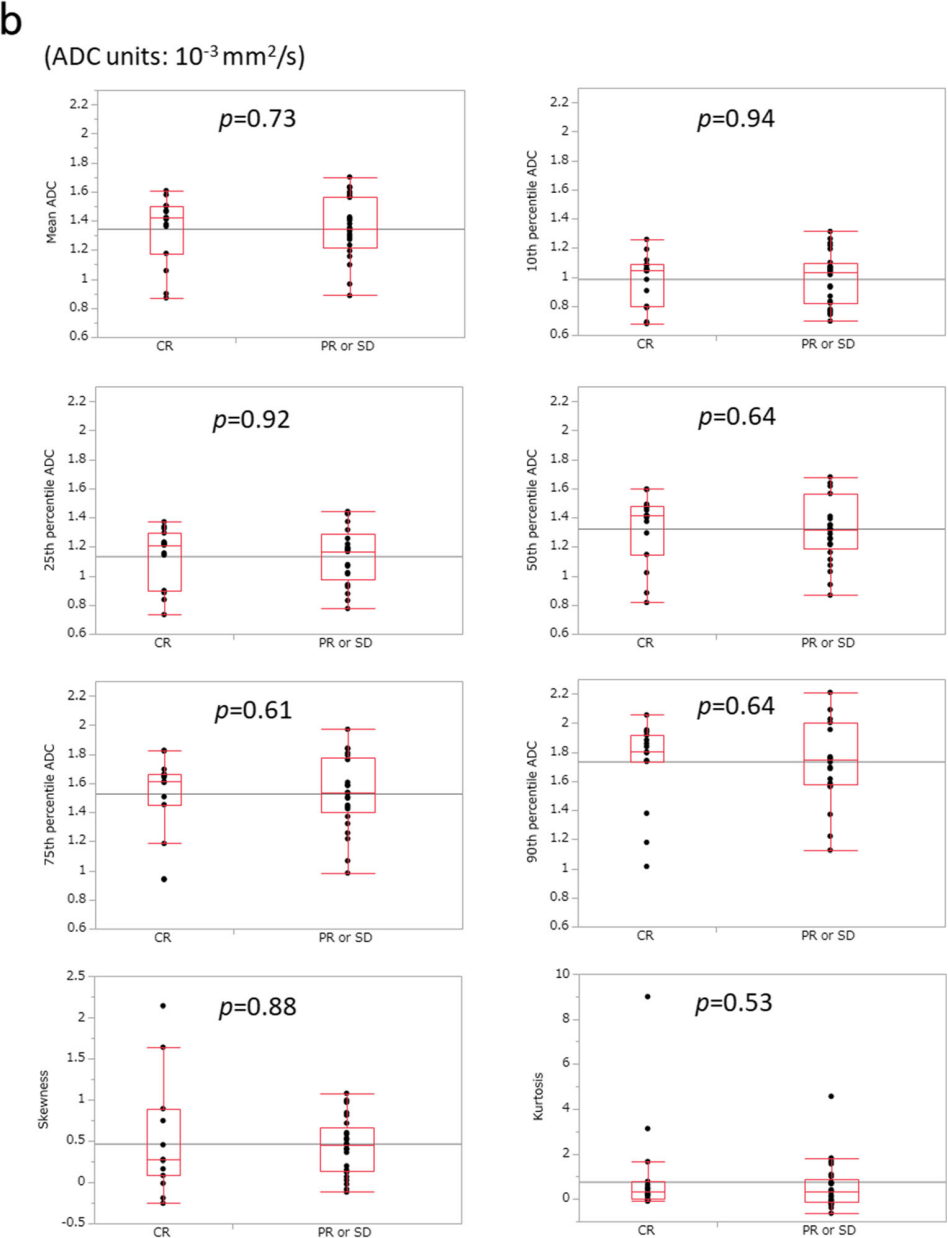
Mean histogram parameters of apparent diffusion coefficient (ADC). Box and whisker plot showing the mean histogram parameters of ADC values in patients with complete response (CR) or partial response (PR) vs. those with stable disease (SD) (**a**) and in patients with CR vs. those with PR or SD (**b**)

The mean values of the largest tumour diameter on pre- and post-treatment CT images were 32.0 ± 8.9 mm and 21.1 ± 8.6 mm, respectively (*p* < 0.001). Post-treatment status was CR in 15 patients (37.5%), PR in 16 patients (40%) and SD in 9 patients (22.5%). No patient was categorized as PD. The mean tumour regression ratio was 34.4 ± 18.9%. The mean number of pixels covering the entire primary lesion on ADC maps was 1339.3 ± 1177.4 (range, 140–3796). Mean histogram parameters of ADC values in patients with CR or PR versus those with SD and in those with CR versus those with PR or SD are shown in Fig. 2 and Table 2. None of the ADC parameters was significantly correlated with post-treatment status (range of Spearman’s ρ values − 0.19 to 0.14, range of *p* values 0.22–0.47) or tumour regression ratio (Spearman’s ρ values − 0.045 to 0.18, range of *p* values 0.26–0.96). There was also no significant correlation between any of the patients’ characteristic data (sex, age and clinical T stage) and treatment response.

**Table 2:** Mean histogram parameters of ADC values in patients with CR or PR vs. those with SD and in patients with CR vs. those with PR or SD

Group	ADC value (×10^−3^ mm^2^/s)					
	CR or PR group	SD group	*p*	CR group	PR or SD group	*p*
Mean	1.36 ± 0.22	1.29 ± 0.22	0.31	1.34 ± 0.23	1.35 ± 0.21	0.73
10th percentile	1.00 ± 0.17	0.94 ± 0.19	0.41	0.99 ± 0.18	0.99 ± 0.18	0.94
25th percentile	1.15 ± 0.19	1.08 ± 0.20	0.37	1.14 ± 0.20	1.14 ± 0.19	0.92
50th percentile	1.34 ± 0.23	1.26 ± 0.22	0.22	1.32 ± 0.24	1.32 ± 0.22	0.64
75th percentile	1.54 ± 0.26	1.47 ± 0.25	0.28	1.52 ± 0.28	1.53 ± 0.25	0.61
90th percentile	1.75 ± 0.28	1.68 ± 0.27	0.39	1.73 ± 0.30	1.74 ± 0.27	0.64
Skewness	0.45 ± 0.50	0.52 ± 0.43	0.47	0.52 ± 0.66	0.43 ± 0.35	0.88
Kurtosis	0.78 ± 1.83	0.73 ± 0.80	0.37	1.12 ± 2.33	0.56 ± 1.06	0.53

**Fig. 3:**
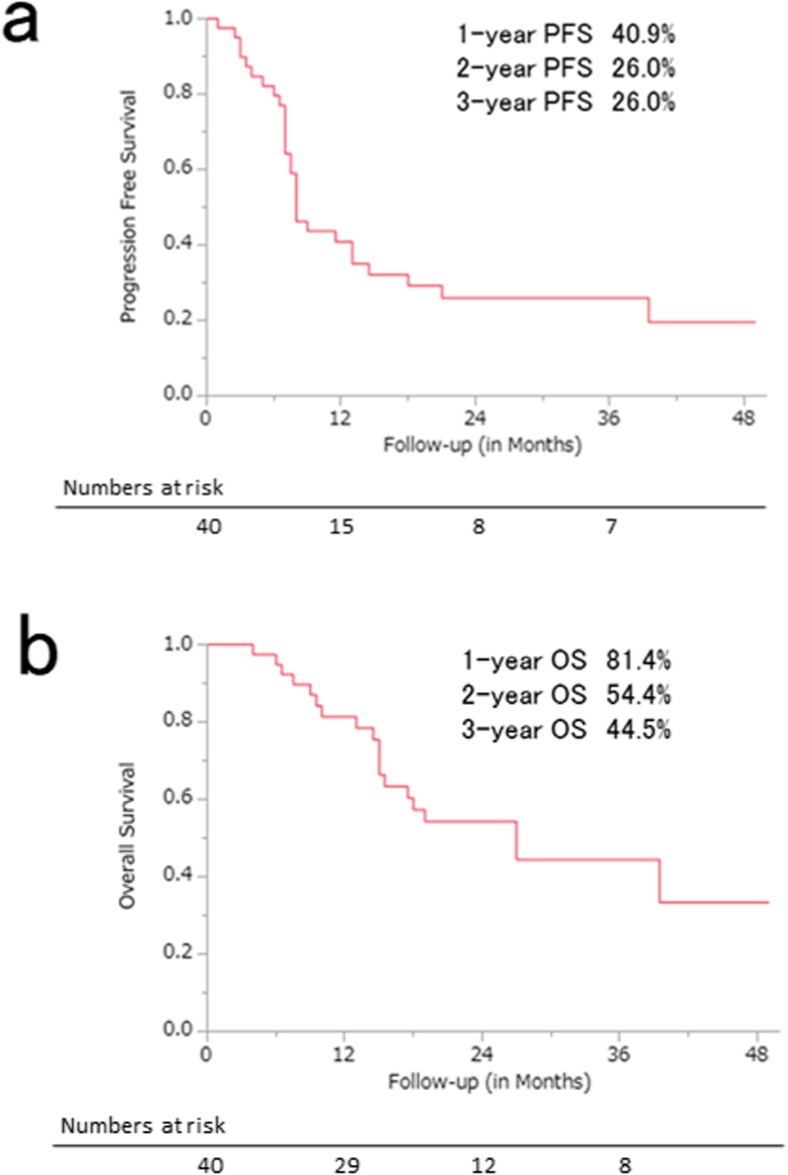
**a** Progression-free survival (PFS) and **b** overall survival (OS) curves for the 40 patients with oesophageal squamous cell carcinoma treated with concurrent chemoradiotherapy

Kaplan–Meier curves for PFS and OS are shown in Fig. 3. The median follow-up periods of all patients and the survivors were 17 months (range, 2–49 months) and 22 months (range, 2–49 months), respectively. The PFS rates at 1, 2 and 3 years in all patients were 40.9% [95% CI 25.0–56.4%], 26.0% [95% CI 12.0–40.4%] and 26.0% [95% CI 12.0–40.4%], respectively, and the OS rates at 1, 2 and 3 years in all patients were 81.4% [95% CI 68.9–93.9%], 54.4% [95% CI 37.7–71.0%] and 44.5% [95% CI 26.0–62.9%], respectively. Eleven patients showed local recurrences and 18 patients showed distant metastases. Three of 11 patients who relapsed underwent surgical resection at a median time of 7 months (range, 7–10 months) after CRT.

**Fig. 4:**
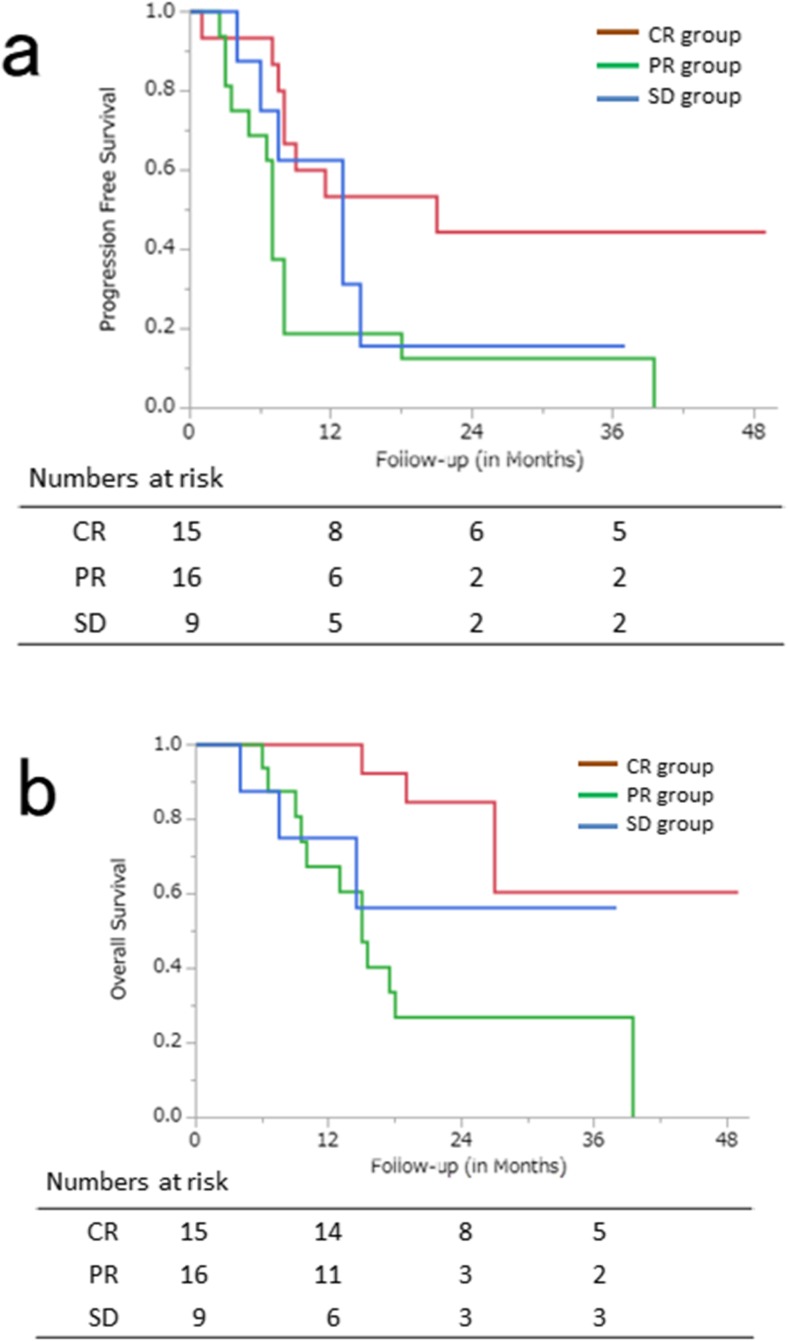
**a** Progression-free survival (PFS) and **b** overall survival (OS) curves according to tumour response. The Kaplan–Meier method and the log-rank test were used for comparison. Survival probabilities are shown as red lines (CR group), green lines (PR group) and blue lines (SD) (*p* = 0.0162 and 0.0095, respectively)

Kaplan–Meier curves for PFS and OS according to post-treatment disease status based on RECIST (CR, PR and SD) are shown in Fig. 4. There were significant differences between patients with CR, those with PR and those with SD in PFS (*p =* 0.02) and OS (*p =* 0.01).

**Fig. 5:**
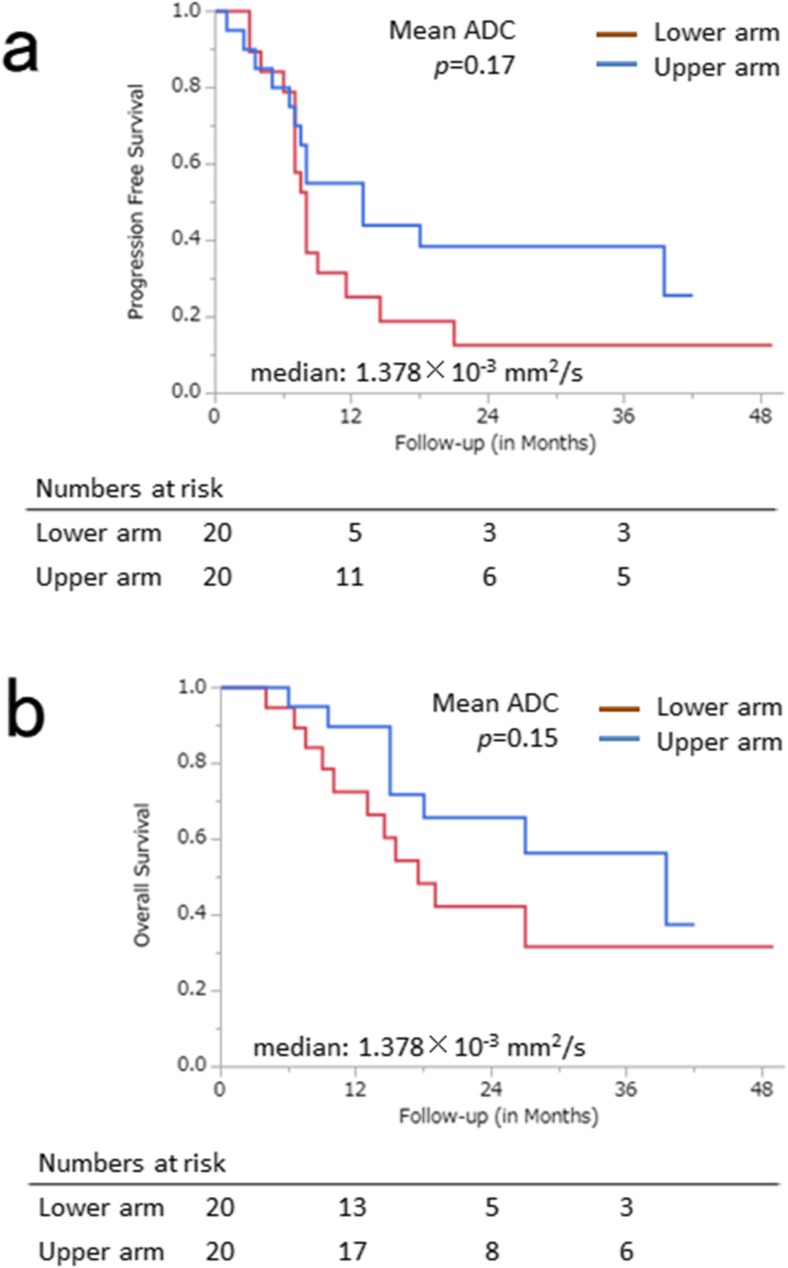
**a** Progression-free survival (PFS) and **b** overall survival (OS) curves obtained by setting the cut-off values to the median values of the separate ADC parameters for the 40 patients with oesophageal squamous cell carcinoma treated with concurrent chemoradiotherapy

Kaplan–Meier curves for PFS and OS, which were obtained by setting the cut-off values to the median values of the ADC parameters, are shown in Fig. 5 and Supplementary Fig. 1. Neither PFS (*p =* 0.17) nor OS (*p =* 0.15) was significantly different between the two groups in the lower (< median) and upper arms ((≥ median) of mean ADC values.

## Discussion

The current study evaluated whether histogram-derived ADC parameters were correlated with tumour response to definitive CRT using readout-segmented echo-planar imaging at 3 T in patients with oesophageal SCC. However, none of the histogram parameters showed significant correlation. There have been a few studies in which the clinical value of DWI for evaluating oesophageal carcinoma was investigated. Aoyagi et al. [13] reported that ADC values of primary tumours decreased as the tumour advanced in stage in 123 patients with oesophageal SCC. The same group [1] also showed that higher mean pretreatment tumour ADC values were associated with longer overall survival and better response to chemoradiotherapy in 80 patients with oesophageal SCC. Wang et al. [10] reported that the series of ADC values in the entire primary lesion were consistently characterised as higher for the CR group compared with those for the PR group at different time points (at the beginning and every week during the course of concurrent CRT) in 38 patients with oesophageal SCC. De Cobelli et al. [23] conducted a study in 32 patients with gastro-oesophageal carcinoma (6 patients with SCC and 26 patients with adenocarcinoma) and showed that pre-neoadjuvant treatment ADC values in responders were lower than those in non-responders. They assessed the therapeutic effect according to histological tumour regression grade. Their results were in conflict with the results obtained by Aoyagi et al. [1] and Wang et al. [10]. We used RECIST to evaluate therapeutic effects, as in the study by Aoyagi et al. [1] and Wang et al. [10]. However, our results are not in line with those reported by Aoyagi et al. and Wang et al. For the ADC parameters, Aoyagi et al. [1] analysed only the average ADC value of the oesophageal carcinoma, while we included the entire primary lesion in the ROI to evaluate the results of histogram analysis. Wang et al. [10] analysed only the average ADC value of the entire primary lesion, but they did not use histogram analysis. In our results, pretreatment ADC values derived from *b*-50 and *b*-800 values were not predictive for oesophageal SCC outcome after response to definitive chemoradiotherapy. Wang et al. [10] used *b*-0 and *b*-600 values and Li et al. [8] used *b*-0 and *b*-700 values for ADC calculations. These different parameter settings might have contributed to the controversy over the usability of diffusion-weighted imaging in predicting response and/or prognosis in patients with oesophageal SCC.

Kwee et al. [24] showed that semi-automated volumetric ADC measurements had higher reproducibility than did manual ADC measurements in monitoring the response to neoadjuvant chemoradiotherapy in patients with oesophageal adenocarcinoma. Several studies indicated high interobserver reliability for measurements of histogram-derived ADC parameters in primary malignant tumours of various organs [7, 25, 26]. Combining our results with those of Aoyagi et al. [1] and De Cobelli et al. [23], it is still debatable whether pretreatment ADC values are predictors of response or prognosis in patients with oesophageal squamous cell carcinoma.

DWI of oesophageal diseases is challenging because of the susceptibility artefacts occurring around air–tissue interfaces and motion artefacts related to respiration, peristalsis and cardiac motion. We applied readout-segmented echo-planar DWI, which generates high-resolution diffusion-weighted images with significantly fewer susceptibility artefacts in comparison with the single-shot technique. Therefore, artefacts on DWI were significantly minimised for ADC mapping of clinical T3–4 oesophageal SCC.

The present study has some limitations. First, our study population was relatively small and the follow-up period was not sufficient to evaluate the predictive value for survival. In the future, it is necessary to perform reanalysis with a longer observation period. Second, it is difficult to accurately measure the primary site of oesophageal carcinoma as distinct from the normal oesophageal wall in one dimension, because CT detection of the primary lesion of oesophageal carcinoma is based on the wall thickness of the oesophagus [27, 28]. Since we did not perform surgical resection within 3 months after CRT in our patients, histological evaluation for treatment response with only biopsy might not be accurate because of sampling errors. Therefore, the therapeutic effect was assessed according to RECIST. De Cobelli et al. [23] reported that gastro-oesophageal cancer was not “measurable” by RECIST, so theoretically it could not be assessed by those criteria; moreover, one-dimensional measurement of gastric wall thickness was critically dependent on stomach distension during the examination. Third, different chemotherapy regimens were used in the patients. It was necessary to tailor the regimen in consideration of each patient’s conditions such as advanced age, heart failure and poor renal function.

In conclusion, our results suggest that pretreatment ADC parameters obtained from readout-segmented echo-planar DWI are not correlated with tumour response to CRT or prognosis in patients with oesophageal SCC, and at least pretreatment ADC measurements alone are not recommended for prediction of the tumour response.

Further investigation that includes long-term follow-up is needed to evaluate the associations between tumour characteristics determined by ADC and patients’ prognoses.

## Electronic supplementary material

ESM 1 (DOCX 412 kb)

## Abbreviations

5-FU: 5-fluorouracil
ADC: Apparent diffusion coefficient
CR: Complete response
CRT: Chemoradiotherapy
DWI: Diffusion-weighted imaging
EPI: Echo-planar imaging
OS: Overall survival
PD: Progressive disease
PFS: Progression-free survival
PR: Partial response
RECIST: Response evaluation criteria in solid tumours
RESOLVE: Readout segmentation of long variable echo-trains
ROI: Region of interest
SCC: Squamous cell carcinoma
SD: Stable disease
SNR: Signal-to-noise ratio
TE: Time of echo
