# Supplementary material for: Oesophageal squamous cell carcinoma: histogram-derived ADC parameters are not predictive of tumour response to chemoradiotherapy
Source: Eur Radiol. 2018 May 3;28(10):4296–305. doi: 10.1007/s00330-018-5439-6 (PMC6132721; doi:10.1007/s00330-018-5439-6)
Supplement: Supplementary file 1 — (DOCX 412 kb) [file 330_2018_5439_MOESM1_ESM.docx]

**
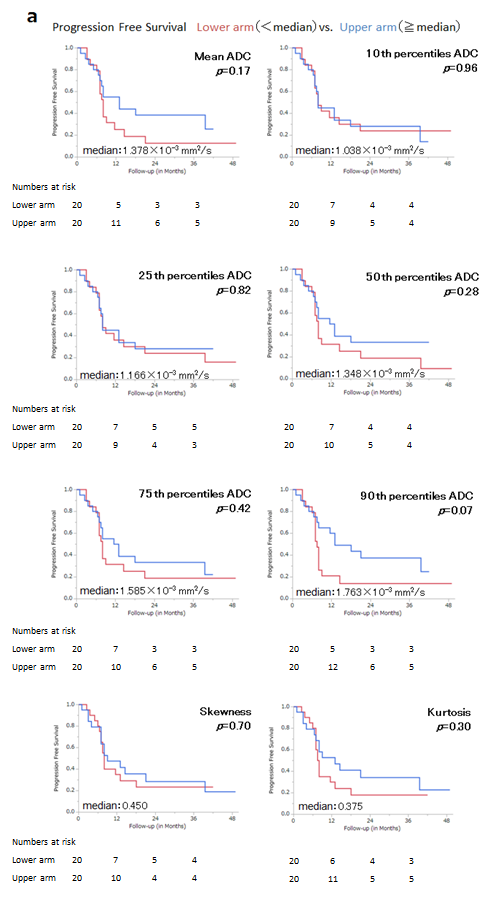

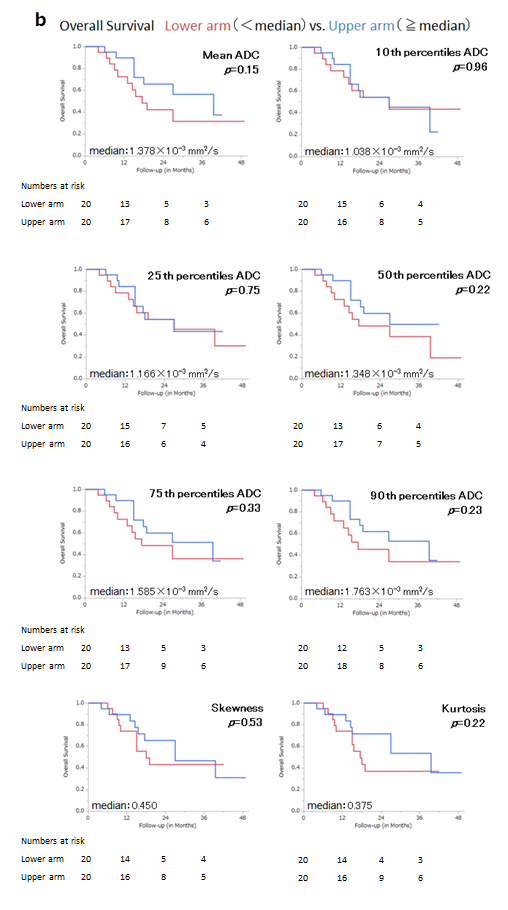
Supplementary Fig.1.** (a) Progression-free survival (PFS) and (b) overall survival (OS) curves obtained by setting the cut-off values to the median values of mean ADC parameters for the 40 patients with esophageal squamous cell carcinoma treated with concurrent chemoradiotherapy.
